# Development of loop-mediated isothermal amplification coupled with nanoparticle-based lateral flow biosensor assay for *Mycoplasma pneumoniae* detection

**DOI:** 10.1186/s13568-019-0921-3

**Published:** 2019-12-05

**Authors:** Yacui Wang, Yi Wang, Weiwei Jiao, Jieqiong Li, Shuting Quan, Lin Sun, Yonghong Wang, Xue Qi, Xingyun Wang, Adong Shen

**Affiliations:** Key Laboratory of Major Diseases in Children, Ministry of Education, National Key Discipline of Pediatrics (Capital Medical University), National Clinical Research Center for Respiratory Diseases, Beijing Key Laboratory of Pediatric Respiratory Infection Diseases, Beijing Pediatric Research Institute, Beijing Children’s Hospital, Capital Medical University, Beijing, 100045 China

**Keywords:** *Mycoplasma pneumoniae*, Loop-mediated isothermal amplification, Nanoparticle-based biosensor, Lateral flow biosensor, LAMP-LFB

## Abstract

*Mycoplasma pneumoniae* (MP) is one of the most common pathogens causing respiratory tract infection, especially for community-acquired pneumonia (CAP) in school-age children. There was considerable amount of studies on loop-mediated isothermal amplification (LAMP) assay for MP detection. However, the result interpretation of these developed LAMP assays was sophisticated and subjective. Therefore, we developed and evaluated a LAMP coupled with nanoparticle-based lateral flow biosensor (LFB) assay (LAMP-LFB) for simple, reliable, and objective identification of MP (MP-LAMP-LFB). Six primers specific to P1 gene of MP were designed, and the preferred temperature for this assay was confirmed to be 65 °C. The amplification products could be visually interpreted by LFB within 2 min. The MP-LAMP-LFB assay specifically identified DNA templates of MP, and no cross-reactivity with other pathogens was obtained. The limit of the detection for this assay was 600 fg of DNA templates in pure cultures, which was in complete accordance with colorimetric indicator detection and agarose gel electrophoresis analysis. This assay was applied to 209 oropharyngeal swab specimens collected from children with acute respiratory tract infection for clinical evaluation, and compared to real-time PCR detection. Using the LAMP-LFB and real-time PCR assay, the positive rates of MP were 47.8% and 31.6%, respectively. Results suggested that the LAMP-LFB assay displayed high sensitivity compared to real-time PCR method. In summary, LAMP-LFB assay established here was a simple, objective, and sensitive assay for MP detection, which can be widely applied in clinical settings, especially in rural areas.

## Introduction

*Mycoplasma pneumoniae* (MP) is one of the leading causes of community acquired pneumonia (CAP) of all ages, especially in school-age children (Marston et al. 1997). MP was responsible for 40% of cases of CAP in children, and as many as 18% of patients requiring hospitalization (Waites and Talkington 2004). Manifestations of MP infection was ordinarily mild and asymptomatic, however, up to 25% of patients may experience extrapulmonary complications, including encephalitis, dermatological disorders, asthma, hemolytic anemia, etc. (Waites et al. 2017; Yis et al. 2008). It was difficult to confirm MP infection for clinicians just through clinical presentations, as it often could be seen with other common pathogens, also treatment with β-lactam antibiotics routinely used for respiratory infections was usually ineffective (Cunha 2006). Thus, laboratory test is of great importance to implement the correct medication strategy for MP infection (Principi and Esposito 2013).

Detection of MP can be achieved by three available methods, including culture-based method, serological assay and nucleic acid amplification technology. Isolation of MP was still the gold standard for definite diagnosis of MP infection (Ozaki et al. 2007). However, culture-based method for MP detection is time-consuming and insensitive, and thus not recommended for conventional diagnosis in clinical settings. Serological test for MP infection has been the cornerstone of MP diagnosis because of the simple and convenient nature of serology. A fourfold or greater rise in antibody of acute- and convalescent-phase sera collected 2 weeks apart was also reliable for MP identification, but it is too slow for early diagnosis in practical application (Kishaba 2016). Comparing with traditional culture-based methods, nucleic acid amplification techniques, such as conventional PCR and real-time PCR, which are fast, sensitive and specific, have been widely used for MP detection (Law et al. 2015). However, PCR-based tests relied on sophisticated instruments conducted by experienced personnel, which are not applicable in rural areas (Zhao et al. 2015). Loop-mediated isothermal amplification method (LAMP), a simple isothermal amplification test with high sensitivity and specificity developed by (Notomi et al. 2000), which has been successfully applied to MP identification (Zhao et al. 2013; Ratliff et al. 2014; Petrone et al. 2015; Yuan et al. 2018). However, the interpretation of LAMP result depends on complicated instruments (real-time turbidimeter), laborious process (agarose gel electrophoresis) and special reagents (colorimetric indicator), which were subjective and limited its application for routine diagnosis.

To achieve better interpretation of the result of LAMP assay, we provided a simple and objective assay, termed as LAMP combined with nanoparticle-based lateral flow biosensor (LFB) assay (LAMP-LFB) for MP detection (Wang et al. 2017). In this report, the LAMP-LFB assay established here was successfully applied for sensitivity and specificity analysis in pure culture of MP, and the clinical specimens was accurately detected by this assay.

## Methods

### Reagents and apparatus

Nanoparticle-based lateral flow biosensor, Isothermal amplification kit, Visual Detection Reagent (VDR) were purchased from BeiJing-HaiTaiZhengYuan Technology Co., Ltd (Beijing, China). QIAamp DNA Mini Kits were purchased from Qiagen Co., ltd (Beijing, China). Primers used in this report were synthesized by Tianyi Huiyuan Biotechnology Co., Ltd (Beijing, China). LA-320C Realtime Turbidimeter was purchased from Eiken Chemical Co., Ltd (Tokyo, Japan). Gel Doc XR + Imaging System was purchased from Bio-Rad Co., Ltd (USA). PCR instrument was purchased from Dongsheng Innovation Biotechnology Co., Ltd (Beijing, China).

### Bacterial strains and clinical samples

To evaluate sensitivity and specificity of LAMP-LFB assay, genomic DNA extracted from 46 reference strains including 20 clinical isolates of MP and 26 strains of non-MP were used for MP detection (Table 1). Oropharyngeal swab specimens were collected from 209 children with acute respiratory infection from October to November in 2018 from Beijing children’s hospital. Children were considered acute respiratory infection highly suspected of MP infection if they had clinical presentations (fever, persistent cough, productive sputum, dyspnea), and/or abnormal laboratory tests, and/or radiographic findings (consolidation, interstitial changes, pleural effusion). Genomic DNA were extracted using QIAamp DNA Mini Kit, and then stored at − 80 °C before use.

**Table 1:** Bacterial strains used in this study

Bacteria	Strain no. (source of strains)	No. of strains	MP-LAMP-LFB
*Mycoplasma pneumoniae*	M129 (BCH)	1	P
	Isolated strains (BCH)	20	P
*Mycoplasma genitalium*	Isolated strain (BCH)	1	N
*Mycoplasma hominis*	Isolated strain (CDC)	1	N
*Mycoplasma penetrans*	Isolated strain (CDC)	1	N
*Mycoplasma primatum*	Isolated strain (CDC)	1	N
*Ureaplasma urealytieum*	Isolated strain (CDC)	1	N
*Chlamydia trachomatis*	Isolated strain (BCH)	1	N
*Bacillus pertussis*	Isolated strain (BCH)	1	N
*Klebsiella pneumoniae*	Isolated strain (BCH)	1	N
*Mycobacterium tuberculosis*	Isolated strain (BCH)	1	N
*Mycobacterium avium*	Isolated strain (BCH)	1	N
*Streptococcus pneumoniae*	Isolated strain (BCH)	1	N
*Pseudomonas aeruginosa*	Isolated strain (BCH)	1	N
*Staphylococcus aureus*	Isolated strain (BCH)	1	N
*Haemophilus influenzae*	Isolated strain (BCH)	1	N
*Stenotrophomonas maltophilia*	Isolated strain (BCH)	1	N
*Acinetobacter baumannii*	Isolated strain (BCH)	1	N
*Legionella pneumophila*	Isolated strain (BCH)	1	N
*H1N1 influenza*	Isolated strain (BCH)	1	N
*H3N2 influenza*	Isolated strain (BCH)	1	N
*H5N1 influenza*	Isolated strain (BCH)	1	N
*H7N9 influenza*	Isolated strain (BCH)	1	N
*Enterococcus faecium*	Isolated strain (BCH)	1	N
*Enterococcus faecalis*	Isolated strain (BCH)	1	N
*Vibrio cholerae*	Isolated strain (BCH)	1	N
*Vibrio vulnificus*	Isolated strain (BCH)	1	N
*Staphylococcus saprophyticus*	Isolated strain (BCH)	1	N

M129, *Mycoplasma pneumoniae* reference strains; BCH, Beijing Children’s Hospital; CDC, Chinese Center for Disease Control and Prevention; P, positive; N, negative

### Primer design

**Fig. 1:**
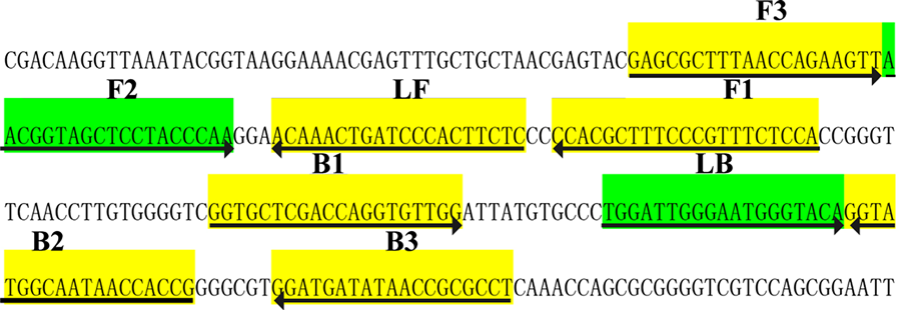
Primers specific to P1 gene of MP used for LAMP-LFB assay. Locations and sequences of the LAMP-LFB primers specific to MP were displayed. The arrows indicated the direction of the primer from 5′ to 3′

A set of six primers, including two outer primers (F3 and B3), two inner primers (FIP and BIP), two loop primers (LF and LB), was designed using two software (Primer Explorer V4 and Primer Premier 5.0) based on P1 gene, which was specific to MP. The specificity of the primers was confirmed by sequence alignment analysis in NCBI database. For MP detection by LAMP-LFB assay, FIP and LF were labeled with FITC and biotin at the 5′ end, respectively. FIP consists of two sequences, a complementary sequence (F1) and a sense sequence (F2). BIP consists of two sequences, a complementary sequence (B1) and a sense sequence (B2). The primer’s sequences, locations and modifications were shown in Fig. 1 and Table 2.

**Table 2:** Primers used in this study

Assay	Primers	Sequence (5′–3′)	Gene
	F3	GAGCGCTTTAACCAGAAGTT	P1
	B3	AGGCGCGGTTATATCATCC	
	FIP*	5′-FITC-TGGAGAAACGGGAAAGCGTGGAACGGTAGCTCCTACCCAA-3′	
LAMP-LFB	BIP	GGTGCTCGACCAGGTGTTGG-CGGTGGTTATTGCCATACC	
	LF*	5′-Biotin-GAGAAGTGGGATCAGTTTGT-3′	
	LB	TGGATTGGGAATGGGTACA	

FITC, fluorescein isothiocyanate; FIP*, 5′-labeled with FITC; LF*, 5′-labeled with biotin

### The standard LAMP-LFB assay

The standard LAMP-LFB reactions with a total volume of 25 μl were prepared, including 24 μl mixed reagents and 1 μl DNA templates, which led to a final concentration of 0.2 mM each of F3 and B3, 1.6 mM each of FIP* and BIP, 0.8 mM each of LF* and LB, 20 mM Tris–HCl (PH = 8.8), 10 mM KCl, 8 mM MgSO_4_, 10 mM (NH4)_2_SO_4_, 0.1% Tween 20, 0.8 mM Betaine, 1.4 mM dNTPS, 8 U/ml Bst DNA polymerase (2 U). In addition, 1 μl genomic DNA of non-MP strains and 1 μl double distilled water were selected as negative control and blank control, respectively. The reactions were performed at 65 °C for 1 h, then the amplification products of the LAMP assay were detected by colorimetric indicator, agarose gel electrophoresis and LFB.

### The optimal amplification temperature for LAMP-LFB assay

To determine the preferred reaction temperature of LAMP-LFB assay for MP detection, we tested the reaction temperature from 60 to 67 °C at 1 °C increments for 1 h, and the results were analyzed using Real-time turbidimeter. Mixtures with 1 μl genomic DNA (600 pg/μl) of MP reference strain (M129), 1 μl *Streptococcus pneumoniae* DNA template, and 1 μl double distilled water were selected as positive control, negative control, and blank control, respectively. The following reactions were conducted at optimal conditions verified above.

### Analytical sensitivity and specificity of LAMP-LFB assay in pure culture

To evaluate the analytical sensitivity of LAMP-LFB assay, serial dilutions (60 ng/μl, 6 ng/μl, 600 pg/μl, 60 pg/μl, 6 pg/μl, 600 fg/μl, 60 fg/μl, 6 fg/μl) of M129 DNA template in pure culture were prepared to confirm the detection limit of the LAMP-LFB assay, and each dilutions were repeated three times. The amplification products were detected by LFB, colorimetric indicator and agarose gel electrophoresis. For specificity assessment of LAMP-LFB assay, DNA template extracted from 26 non-MP strains were used. Each specimen was tested at least twice.

### Application of LAMP-LFB assay in clinical specimens

To assess the applicability of LAMP-LFB assay in clinical samples, oropharyngeal swab specimens collected from 209 children with acute respiratory infections highly suspected of MP infection were tested by LAMP-LFB assay. The results of LAMP-LFB assay for MP identification were compared with that of real-time PCR for the same samples. Real-time PCR were conducted with *Mycoplasma pneumoniae* and Macrolide-Resistant isolates Diagnostic Kits, which were purchased from Mole BioScience Co., Ltd (Jiangsu, China).

## Results

### Confirmation and detection of amplification of MP-LAMP assay

**Fig. 2:**
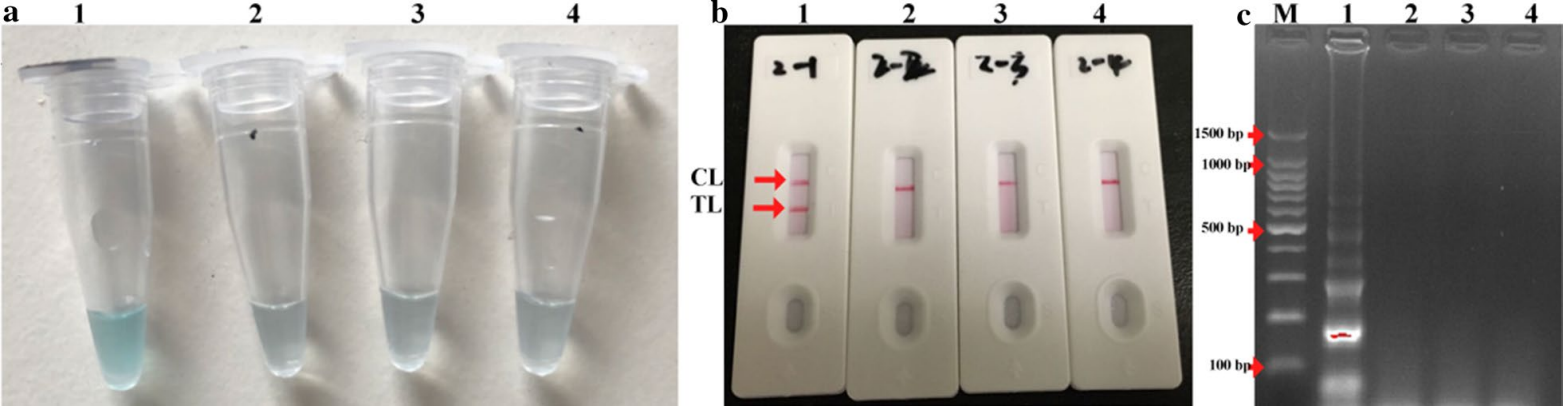
Confirmation and detection of MP-LAMP amplification products. **a** Amplification products of MP-LAMP assay were visually detected by colorimetric indicators. **b** LFB applied for visual identification of MP-LAMP products. **c** Amplification products of MP-LAMP assay were detected by agarose gel electrophoresis. Tube/biosensor/lane: (1) positive products of MP; (2) negative control of *Streptococcus pneumoniae*; (3) negative control of *Staphylococcus aureus*; (4) blank control of double distilled water

In order to assess the availability of the primers designed for LAMP assay, LAMP reactions were conducted in the presence and absence of MP DNA template at 65 °C for 1 h. The amplification products were monitored by three detection methods, including agarose gel electrophoresis, colorimetric indicator and LFB. The positive amplification detected by colorimetric indicator was visualized as light green, while the negative and blank controls remained colorlessness (Fig. 2a). Using agarose gel electrophoresis, many bands of different sizes displayed typical ladder-like pattern in the positive LAMP products, but not in the negative and blank controls (Fig. 2c). By LFB, two red lines (test line, TL; control line, CL) were shown in the positive reactions, whereas only one red line (CL) was exhibited in the negative and blank controls (Fig. 2b). These results demonstrated that the MP-LAMP primer set designed here was able to specifically amplify the target sequence and can be used as a good candidate for establishment of LAMP-LFB assay for MP detection.

### The optimal reaction temperature of LAMP assay

**Fig. 3:**
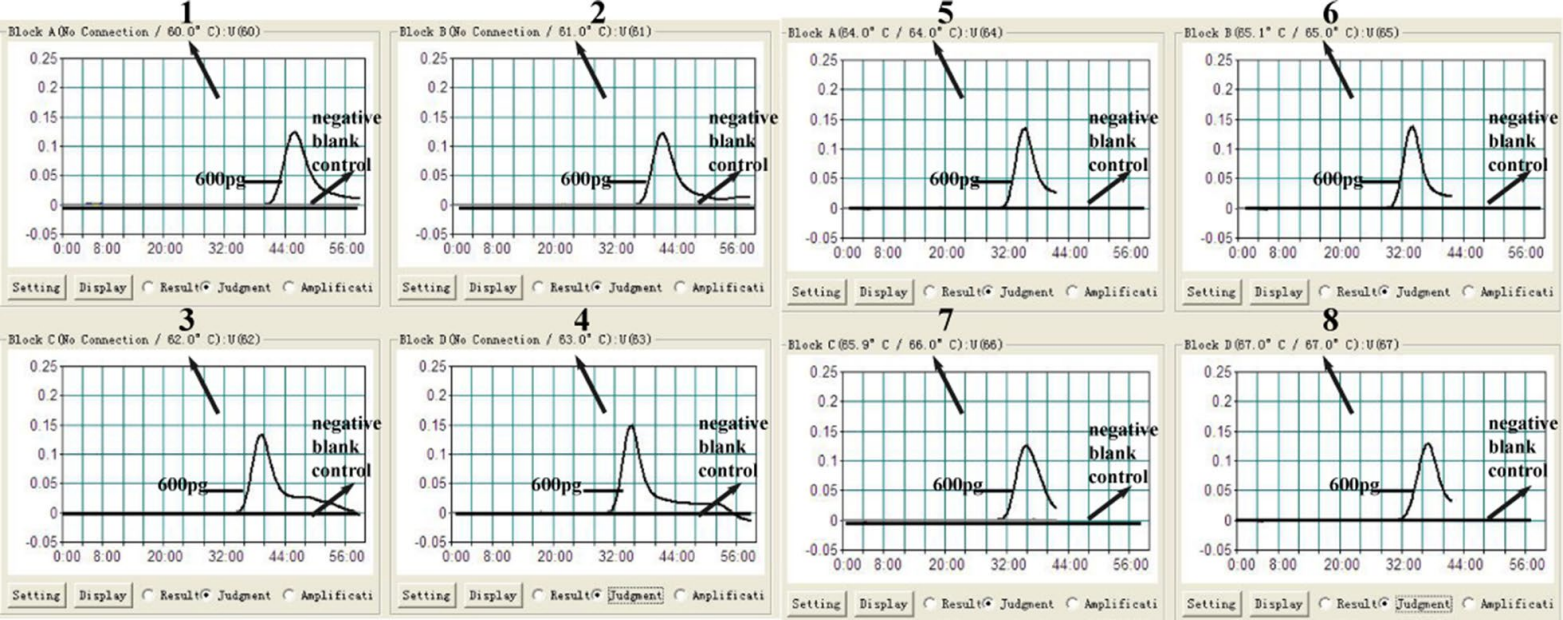
Optimal reaction temperature of MP-LAMP assay. The amplification products of MP-LAMP assay were monitored by Real-time turbidimeter, and the corresponding curves of each temperature were displayed in the picture. Turbidity of > 0.1 was considered positive. Eight kinetic graphs (1–8) were acquired at different temperatures ranging from 60 to 67 °C with the concentration of DNA templates of MP at the level of 600 pg/ul

To determine the preferred temperature of LAMP assay during amplification stage, LAMP was performed with genomic DNA of MP at the level of 600 pg/μl per reaction at eight distinct temperatures from 60 °C to 67 °C at 1 °C intervals. The amplification products were detected by Real-time turbidimeter, and eight typical kinetic graphs corresponding to each temperature were obtained. As shown in Fig. 3, 65 °C was confirmed to be the preferred reaction temperature with the faster amplification efficiency. Therefore, 65 °C was selected as the optimal temperature for the subsequent LAMP conduction for MP detection in this report.

### Sensitivity and specificity of LAMP-LFB assay

**Fig. 4:**
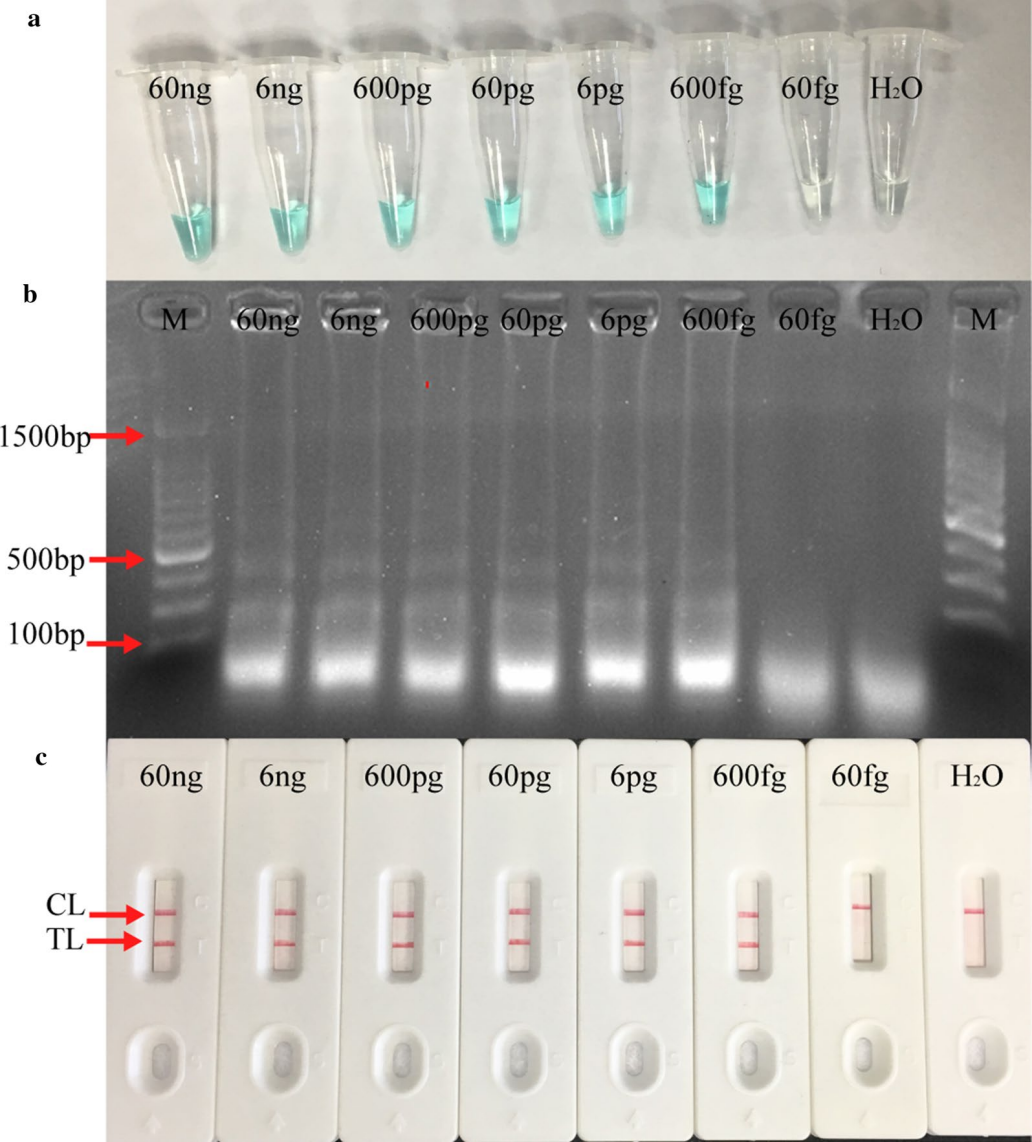
Analytical sensitivity of MP-LAMP assay. Three monitoring methods: **a** colorimetric indicator; **b** agarose gel electrophoresis; **c** LFB were used for amplification products analysis. Serial dilutions (60 ng, 6 ng, 600 pg, 60 pg, 6 pg, 600 fg, 60 fg, 6 fg) of genomic DNA of MP were used for sensitivity determination of LAMP assay. The DNA templates of MP from 60 ng to 600 fg produced positive results

**Fig. 5:**
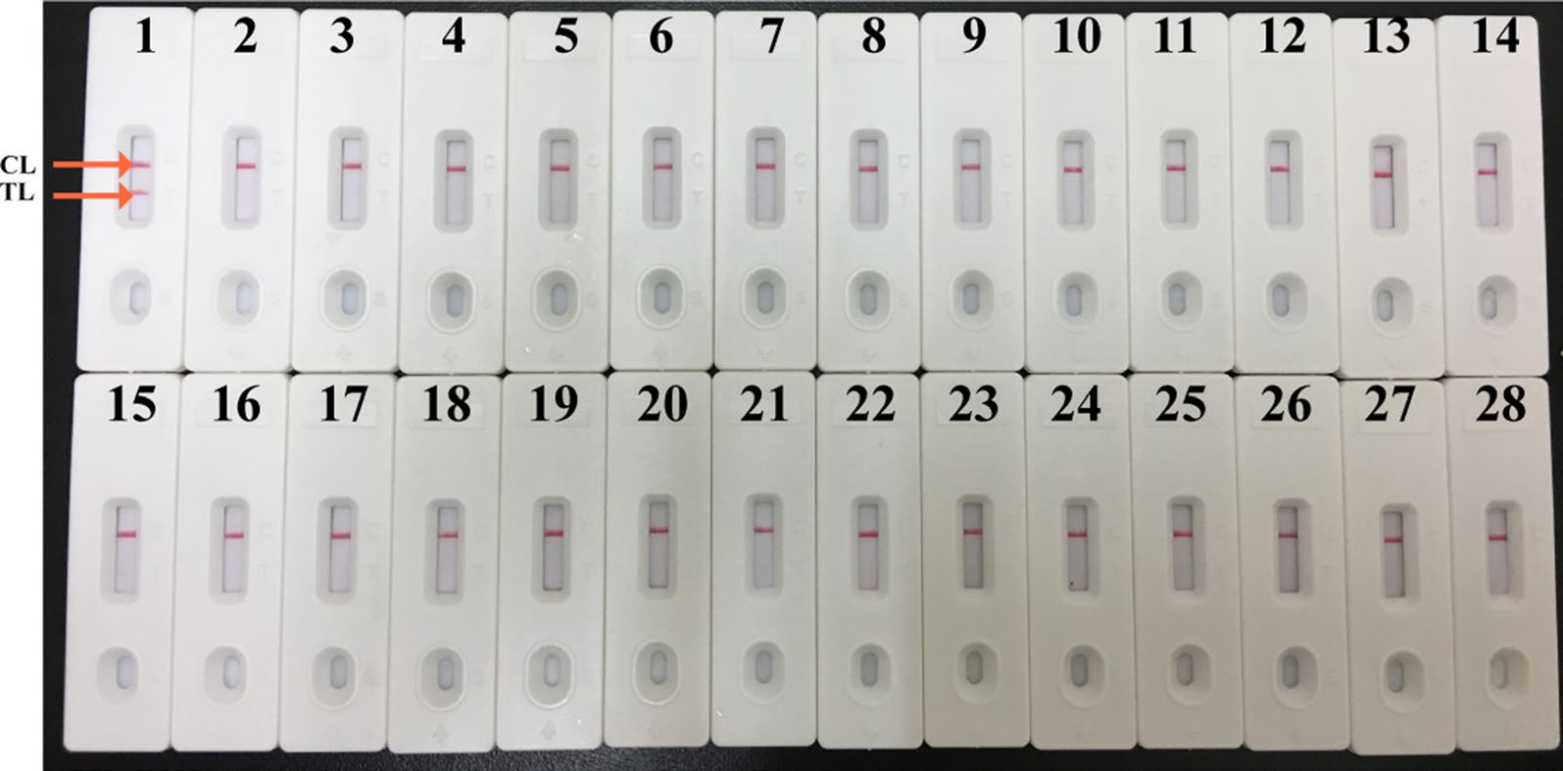
Analytical specificity of MP-LAMP assay. The LAMP reactions were conducted using genomic DNA templates from different pathogens, and were monitored by LFB. Bisensor 1, MP standard strain (M129); biosensors 2-27, *Mycoplasma genitalium*, *Mycoplasma hominis*, *Mycoplasma penetrans*, *Mycoplasma primatum*, *Ureaplasma urealytieum*, *Chlamydia trachomatis*, *Bacillus pertussis*, *Klebsiella pneumoniae*, *Mycobacterium tuberculosis*, *Mycobacterium avium*, *Streptococcus pneumoniae*, *Pseudomonas aeruginosa*, *Staphylococcus aureus*, *Haemophilus influenzae*, *Stenotrophomonas maltophilia*, *Acinetobacter baumannii*, *Legionella pneumophila*, *H1NI influenza*, *H3N2 influenza*, *H5N1 influenza*, *H7N9 influenza*, *Enterococcus faecium*, *Enterococcus faecalis*, *Vibro cholerae*, *Vibrio vulnificus*, *Staphylococcus saprophyticus*; biosensor 28, blank control (double distilled water)

The analytical sensitivity of LAMP-LFB assay for MP detection was determined by analyzing LAMP amplifications of serial dilutions (60 ng, 6 ng, 600 pg, 60 pg, 6 pg, 600 fg, 60 fg, 6 fg) of M129 genomic DNA in triplicate. As shown in Fig. 4, the limit detection (LoD) of LAMP assay was 600 fg by each monitoring method. These results demonstrated that the LoD of LAMP assay for MP detection by agarose gel electrophoresis, colorimetric indicator was conformity with LFB analysis. For specificity evaluation, DNA template extracted from 26 non-MP pathogens were used. No cross-reactivity with other agents apart from MP was observed, which indicated that the LAMP-LFB assay has a 100% specificity for MP detection (Fig. 5).

### Evaluation of the MP- LAMP-LFB assay using clinical specimens

To further verify the availability of MP-LAMP-LFB assay in clinical samples, 209 oropharyngeal swab specimens collected from patients were tested. Of the 209 samples, 100 (47.8%) samples were confirmed to be positive by LAMP-LFB assay, while only 66 (31.6%) samples were detected positive by real-time PCR method. The results demonstrated that MP-LAMP-LFB assay was more sensitive than real-time PCR for MP detection (Table 3).

**Table 3:** Comparison of LAMP-LFB assay and real-time PCR for MP detection

Detection assays	Oropharyngeal swab samples	
	Positive	Negative
LAMP-LFB	100 (47.8%)	109 (52.2%)
Real-time PCR	66 (31.6%)	143 (68.4%)

Data were presented as number (%)

## Discussion

Being easily transmitted by airborne droplets, MP can induce outbreaks in crowded places, especially in the school and community. Accordingly, it’s of great significance to implement treatment with effective antibiotics to end the transmission by earlier diagnosis of MP infection. Current available techniques for MP detection, such as culture-based method, serological test and real-time PCR assay are time-consuming, sophisticated and not suitable for clinical application. To overcome these shortcomings posed by facilities illustrated above, we developed a simple, fast, and reliable assay, LAMP-LFB assay, for MP detection.

In this report, LAMP-LFB assay that targeting P1 gene specific to MP was successfully developed and evaluated. The LoD of this assay was as low as 600 fg per reaction in pure culture (Fig. 4). In addition to its excellent sensitivity, LAMP-LFB assay also presented high specificity for MP identification in 26 other pathogens, and no cross-reactivity was observed expect for MP (Fig. 5), which demonstrated that the LAMP-LFB assay was reliable for the target agent detection. For clinical application evaluation, commercialized real-time PCR assay targeting P1 gene of MP was selected as a control method. Genomic DNA extracted from 209 oropharyngeal swab samples were detected simultaneously by LAMP-LFB assay and real-time PCR technique. The results indicated that LAMP-LFB assay with the detection rate of 47.8% was more sensitive than real-time PCR of 31.6% (Table 3). However, several studies reported that LAMP assay displayed almost the same sensitivity as real-time PCR technique for MP detection (Saito et al. 2005; Yoshino et al. 2008), or was less sensitive than real-time PCR (Zhao et al. 2013; Petrone et al. 2015; Yuan et al. 2018). The LAMP-LFB assay was still an alternative to PCR-based method for the following advantages. First, it was fast with high efficiency. The amplification process could be accomplished in 30 to 60 min, with up to 10^10^ copies of the target gene obtained (Yuan et al. 2018). Second, high specificity was another advantage of LAMP-LFB assay compared to PCR-based method for MP detection, as six primers specific to P1 gene of MP could accurately recognize the corresponding region of the target sequence. Apparatus for LAMP assay was just a heating block that was easily acquired, also it was less expensive than PCR-based method. The economic and simple character of LAMP-LFB assay making it more acceptable to clinicians.

Previously, a large amount of LAMP assays had been successfully established for MP detection, the amplification products were monitored by agar gel electrophoresis, Real-time turbidimeter and colorimetric indicator (Zhao et al. 2013; Yuan et al. 2018; Saito et al. 2005). Agarose gel electrophoresis relied on complicated, expensive apparatus, trained laboratories and personnel, which were not suitable for application in primary hospitals, and so did the Real-time turbidimeter. Though colorimetric indicator was simple and low-priced, it could be somewhat ambiguous when the concentration of the amplicon was low. LFB avoids these shortcomings, easy to operate, and diagnostic determination can be made within 2 min. The performance of LFB for MP detection depends on the primers of FIP and LF, which were labeled with FITC and biotin, respectively. During the amplification process, the positive double-labeled amplification products were formed. One end of the positive products labeled with FITC was captured by the anti-FITC antibody immobilized on the test line (TL) of the biosensor, and the other end of the positive products labeled with biotin were combined with nanoparticles for visualization. The rest of the nanoparticles, which could be seized by the biotinylated bovine serum albumin fixed on the control line (CL) to confirm the availability of the biosensor. The interpretation of amplification products depended on the red bands displayed on the biosensor. Two red lines (TL and CL) appeared on the biosensor indicated positive amplifications of MP, while only one red line (CL) demonstrated negative and blank controls. Considering above, LAMP-LFB assay established here was a good candidate for MP detection.

In conclusion, we successfully established and evaluated the LAMP-LFB assay for MP detection both in pure culture and in clinical samples at 65 °C for 1 h, which has the potential to be a precious test for MP surveillance and detection in the early stage of the infection. The merits of LAMP-LFB assay established here are simplicity, rapidity, sensitivity and specificity. Therefore, the LAMP-LFB assay was a promising method for MP detection in medical institutions, especially in rural areas. A limitation of this study is the limited specimen types. Later, other types of clinical samples will be included to validate the availability of LAMP-LFB assay.

## Data Availability

We declared that materials described in the manuscript, including all relevant raw data, will be freely available to any scientist wishing to use them for non-commercial purposes, without breaching participant confidentiality.

## Abbreviations

MP: *Mycoplasma pneumoniae*
LAMP: loop-mediated isothermal amplification
LFB: nanoparticle-based lateral flow biosensor
CAP: community acquired pneumonia
